# Awareness and willingness to use pre‐exposure prophylaxis among the University of Zambia students: A cross‐sectional study

**DOI:** 10.1002/hsr2.70060

**Published:** 2024-09-09

**Authors:** Martin Kampamba, Natalia N. Nelumbu, Christabel N. Hikaambo, Steward Mudenda, Jimmy M. Hangoma, Mwape Kunda, Webrod Mufwambi, Audrey Hamachila, Moses Mukosha

**Affiliations:** ^1^ Department of Pharmacy School of Health Sciences, University of Zambia Lusaka Zambia; ^2^ Department of Pharmacy School of Health Sciences, Levy Mwanawasa Medical University Lusaka Zambia; ^3^ Department of Pharmacy Mary Begg Health Services Ndola Zambia; ^4^ HIV and Women's Health Research Group University Teaching Hospital Lusaka Zambia; ^5^ Department of Epidemiology and Biostatistics School of Public Health, University of the Witwatersrand Johannesburg South Africa

**Keywords:** pre‐exposure prophylaxis, prevention, students, uptake, willingness

## Abstract

**Background and Aims:**

Despite a quick rollout of PrEP as a preventive method against Human Immunodeficiency Virus (HIV) infections in Zambia, adolescent and young adult populations have remained very vulnerable to HIV infection. This study assessed the awareness and willingness to use PrEP among University of Zambia (UNZA) students.

**Methods:**

Three hundred forty‐six students participated in this cross‐sectional study at UNZA between March and June 2021. A previously validated questionnaire assessed willingness to use PrEP. We tested the hypothesized pathways between sexual risk behavior and willingness to use PrEP using the structural equation model**.** Multivariate logistic regression analysis was employed to determine factors associated with willingness to use PrEP. Variables with a *p*‐value < 0.05 were considered statistically significant.

**Results:**

Of the 346 students, 271 (78.3%) were aware of PrEP, and 59 (17.1%) of the participants were willing to use PrEP. Only 17 (4.9%) of the participants had used PrEP before**.** In the multivariable logistic regression model, students who were aware of PrEP compared to those who were not (AOR = 3.03, 95% CI: 1.10, 8.40, p) were more likely to be willing to use PrEP. Sexual risk behavior indirectly and positively affected willingness to use PrEP through awareness of PrEP.

**Conclusion:**

Even though most students were aware of PrEP, the willingness to use this preventative measure is still low among UNZA students, resulting in low uptake. Therefore, concerted efforts are required to influence the willingness and uptake of PrEP, especially in high‐risk age groups such as university students**.**

## 1 BACKGROUND

In the last three decades, HIV/AIDS has been the leading cause of death among the age group of 15–49 years, especially in the sub‐Saharan region of Africa.^1^, ^2^ HIV/AIDS was responsible for 1.7% of all deaths worldwide in 2018, rising to 28% in Sub‐Saharan Africa.^1^, ^3^ Zambia's HIV epidemic is generalized, with a persistently high prevalence of about 12% and an annual incidence of 0.61% among adults aged 15–59 years old.^4^ Concentrated pockets of high HIV transmission, however, disproportionately affect populations that face substantial psychosocial and structural barriers to accessing health services and continue to drive epidemic transmission.^5^

To prevent HIV in high‐risk adults, daily oral pre‐exposure prophylaxis (PrEP) is recommended.^4^, ^5^ PrEP is a novel biological advance towards HIV prophylaxis, in which people at high risk of contracting HIV infection take oral antiretroviral, typically a combination of tenofovir and emtricitabine, to prevent the infection.^6^ Adherence to PrEP is closely related to its effectiveness, with research indicating that when adherence is >70%, PrEP is most effective.^7^, ^8^, ^9^ Several clinical trials have conclusively shown that a combination of tenofovir disoproxil fumarate and emtricitabine reduces the risk of HIV transmission.^10^, ^11^ The US Food and Drug Administration approved the use of tenofovir disoproxil fumarate and emtricitabine combination as PrEP in 2012 based on these findings.^12^ PrEP has since gained acceptance in several nations, including the United Kingdom, the United States, France, China, and Zambia, among others.^13^ Furthermore, to prevent new HIV infections among HIV‐affected populations, the World Health Organization (WHO) recently recommended the adoption of PrEP.^14^, ^15^ Only a few countries in Sub‐Saharan Africa, including South Africa, Kenya, Uganda, Zimbabwe, and Zambia, have initiated large‐scale national PrEP programs, despite the evidence of its effectiveness and the WHO's recommendation.^16^

Zambia introduced the daily intake of PrEP for free in all public hospitals in 2016 as an additional HIV preventative measure in addition to condom use.^17^, ^18^, ^19^ Since the introduction of PrEP, more than 147, 397 clients had started PrEP by 2021.^2^ Despite using PrEP as part of HIV prevention programs, new HIV infections continue to rise, and the burden of new HIV infections falls disproportionately on adolescents and young adults in Sub‐Saharan Africa.^20^, ^21^ A lack of awareness and willingness to use PrEP could be one of the drivers of the increase in new infections among adolescents and young adults. This is despite the many national programs that nations have used to raise awareness, such as raising demand through the media and community health talks and streamlining PrEP distribution through ART clinics and drop‐in centers for key populations.^17^, ^19^, ^22^

For example, a study conducted in Lesotho among university students revealed that even though 57.3% of participants perceived themselves at risk of acquiring HIV, only 32.1% reported being strongly willing to use PrEP if available in their community.^23^ Another study in Nigeria found 18.9% PrEP awareness among university students. In this study, testing for HIV and knowledge of a partner's HIV status were associated with awareness of PrEP.^24^ A similar study in South Africa also showed a low awareness of 18.8% among university students, with only 1.7% of participants having used PrEP.^25^

A study conducted at Nkrumah University in Zambia revealed a very low knowledge level and a negative attitude towards PrEP among late adolescents and young adults.^26^ In light of the recent rollout of PrEP in Zambia and the lack of evidence on how adolescents perceive its use, there is an urgent need to understand the willingness to use this service among this high‐risk population. Therefore, the current study aimed to determine the factors that affect the willingness to use PrEP among university students. In addition, we examined the pathways linking sexual risk behavior to the willingness to use PrEP among university students.

## 2 METHOD AND MATERIALS

### 2.1 Study design, setting, and population

This was a cross‐sectional study conducted at the University of Zambia (UNZA) in Lusaka between 1st February and 30th April 2021. UNZA is the largest learning institution in Zambia, with over 4000 registered students and over 100 undergraduate learning programs.

To be eligible for this study, students should have been 18 years of age or older, studying full‐time at UNZA, willing to respond to the questionnaire, and have signed a fully informed consent form. Students living with HIV were excluded from the study.

### 2.2 Sampling and sample size considerations

We used the single population proportion formula, and our minimum estimated sample size was 385, considering an estimated proportion of (0.5), Z score at the 95% confidence level with a 5% margin of error. Necessary Sample Size = (Z‐score)^2^ * P*(1‐P)/(margin of error).

Potential participants were identified through the university's student register. Then we randomly selected eligible students using computer‐generated random numbers after stratification by program and year of study. We sent out questionnaires, information sheets, and consent forms through emails with links to google forms@. Participants only had access to the questionnaires after retaining the signed consent forms. The Google form had a screening question, and only those who reported a negative HIV serostatus were able to access the form.

Reminders were sent every week to those who had not responded for at least three consecutive weeks to increase the response rate.

### 2.3 Data collection and study variables

A self‐administered questionnaire adapted from previous studies was used to collect the data.^25^, ^27^ The electronic questionnaire comprised two parts, which permitted the effective capture of structured responses on sociodemographics, sexual behavior, and the level of awareness and willingness of PrEP.

The primary outcome of this study was the willingness to use PrEP, which was measured on a binary scale (coded: yes = 1, no = 0). Students were asked to respond yes or no to the question, “If PrEP was available in my community, I would probably take it.” Additional information was obtained about the student's age, sex, level of study, resident type, living arrangements, religion, and whether they were taking health‐related courses or not. Furthermore, we collected information about the sexual behavior of the respondents. Before collecting data, we delivered the questionnaire to 30 students from a separate university for a pilot survey so that we could fine‐tune the survey questionnaires, methods, and data collection instruments to ensure clarity, unbiasedness, and effectiveness and adjust the data collection instruments to suit our setting. The internal consistency of the questionnaire was tested using Cronbach's alpha, and all the questions were found to be above 0.7, suggesting that the questions were reliable.

**Figure 1:**
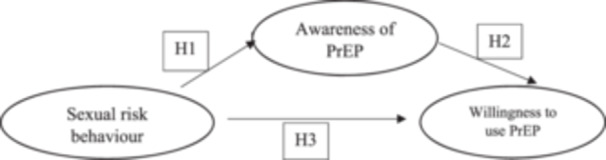
Hypothetical model of the mediating role of pre‐exposure prophylaxis (PrEP) awareness on the willingness to use PrEP.

Based on the available literature,^23^ we hypothesized that the sexual risk behavior of a student would have a direct effect on their willingness to use PrEP. The student's awareness of PrEP would mediate this effect on their willingness to use PrEP. We proposed a model (Figure 1) and assumed that the expected covariance matrix in the hypothesized link does not differ from our sample covariance matrix. Furthermore, we assumed that the sexual risk behavior of a student directly and positively influences both the awareness of using PrEP (H1 > 0) and the willingness to use PrEP among students at UNZA（H3 > 0). In addition, awareness of PrEP directly and positively affects willingness to use PrEP (H2 > 0). Finally, sexual risk behavior positively affects willingness to use PrEP through the student's awareness of PrEP (H1* H2 > 0).

## 3 STATISTICAL ANALYSIS

The analyses were conducted in Stata/BE, version 17 (Stata Corporation, College Station, Texas, USA). The internal consistency of the scale measuring sexual risk behavior was assessed by evaluating Cronbach's alpha. Categorical variables were presented as frequencies (percentages). Bivariate analyses of the outcome (willingness to use PrEP) were assessed against exposure variables: sexual risk behavior, awareness of PrEP, and sociodemographic factors. Pearson Chi‐square test or Fisher's exact test were conducted to examine differences between groups.

The primary analysis assessed the association between sexual risk behavior and willingness to use PrEP, in a logistic regression model. From the univariable models, sociodemographic factors associated with willingness to use PrEP (*p* < 0.2) were adjusted for in the multivariable analysis using investigator‐led model selection techniques until the models appeared parsimonious. In the final model, interactions were assessed and none reached any statistical significance. The Hosmer‐Lemeshow test was used to assess the goodness of fit for the final model.

In an exploratory analysis we fitted a Structural Equation Model (SEM) with maximum likelihood to test the hypothesized pathways between sexual risk behavior, potential mediators (awareness of PrEP), and willingness to use PrEP. Evidence from literature and bivariate analysis was used to inform preliminary model building based on the proposed hypothesis (Figure 1). The model modification was performed based on modification indices and theoretic reasoning. Measures for model goodness of fit included (root mean square error of approximation (RMSEA) and an incremental measure (Bentler's comparative fit index (CFI)) (1). The model fit was adequate based on the criteria RMSEA < 0.05 and CFI ≥ 0.93. All statistical tests are presented at the significance level of alpha <0.05 (two sided test) and 95% confidence intervals.

## 4 ETHICAL APPROVAL

The study was conducted in line with the Declaration of Helsinki (1964) or its subsequent revisions regarding studies with human subjects. The protocol for this study was approved by the University of Zambia School of Health Sciences Research Ethics Committee (Study protocol ID: 20203101050). We obtained informed, written consent from all participants before the study. The participants' confidentiality and anonymity were strictly maintained to the extent legally possible. We referred all the participants who screened with high sexual risk behavior to the psychosocial counselor. Participants were allowed not to answer any questions they were not comfortable with and what they felt were sensitive questions.

## 5 RESULTS

Three hundred sixty out of 385 undergraduate students responded to the questionnaire, giving the response rate of 94.7%. Fourteen students who self‐reported to be HIV positive were excluded from the study, and therefore, data analysis was limited to 346 self‐reported HIV‐negative students. Of the 346 participants, 205 (59.3%) were females. Slightly above a third, 159 (46.0%) were between 18 and 23 years old, and 157 (45.4%) sourced information about PrEP from healthcare workers. A larger proportion, 87 (25.1%) were in their fourth year of study, and 194 (56.1%) were enrolled in a health‐related program. 206 (59.5%) lived with more than one roommate, and 191 (55.2%) lived off‐campus. Roughly four in five, 286 (82.7%) were of the Christian faith, and 271 (78.3%) were aware of PrEP services in Zambia (Table 1). Awareness of PrEP services was significantly associated with willingness to use PrEP among participants (*p* = 0.007).

**Table 1:** Sociodemographic characteristics of study participants and their willingness to use pre‐exposure prophylaxis (PrEP) (*n* = 346).

	Total population	Willingness to use PrEP		
Variables	*n* (%)	No, *n* (%)	Yes, *n* (%)	*p*‐Value
Age (years)				
18‐23	159 (46.0)	137 (86.2)	22 (13.8)	0.20
24‐29	113 (32.7)	88 (77.9)	25 (22.1)	
≥30	74 (21.4)	62 (83.8)	12 (16.2)	
Sex				
Female	205 (59.3)	167 (81.5)	38 (18.5)	0.38
Male	141 (40.8)	120 (85.1)	21 (14.9)	
Year of Study				0.09
First	31 (9.0)	27 (87.1)	4 (12.9)	
Second	77 (22.3)	69 (89.6)	8 (10.4)	
Third	43 (12.4)	37 (86.1)	6 (13.9)	
Fourth	87 (25.1)	74 (85.1)	13 (14.9)	
Fifth	82 (23.7)	60 (73.2)	22 (26.8)	
Sixth	26 (7.5)	20 (76.9)	6 (23.1)	
Program of study				
Non‐health related	152 (43.9)	126 (82.9)	26 (17.1)	0.98
Health‐related	194 (56.1)	161 (83.0)	33 (17.0)	
Residence				
Off‐campus	191 (55.2)	155 (81.2)	36 (18.8)	0.32
On campus	155 (44.8)	132 (85.2)	23 (14.8)	
Living arrangements				
Alone	31 (9.0)	23 (74.2)	8 (25.8)	0.35
One roommate	109 (31.5)	93 (85.3)	16 (14.7)	
>one roommate	206 (59.5)	171 (83.0)	35 (17.0)	
Religion				
Other	60 (17.3)	53 (88.3)	7 (16.7)	0.22
Christianity	286 (82.7)	234 (81.8)	52 (18.2)	
Source of information				
Friends/family	47 (83.0)	39 (17.0)	8 (13.6)	0.38
Health workers	157 (45.4)	128 (81.5)	29 (19.5)	
Nowhere	75 (21.7)	67 (89.3)	8 (10.7)	
Media	67 (19.4)	53 (79.1)	14 (20.9)	
Aware of PrEP				
No	75 (21.7)	70 (93.3)	5 (6.7)	0.007
Yes	271 (78.3)	217 (80.1)	54 (19.9)	
Overall		59 (17.1)	287 (82.9)	

*Note*: Results are frequency (%). The differences in proportions was assessed using Pearson Chi‐square test or Fisher's exact test as appropriate.

Abbreviation: PrEP, pre‐exposure prophylaxis.

Table 2 shows the cross‐tabulation of sexual risk behavior indicators and willingness to use PrEP. The majority, 261 (75.4%), reported having sex before, 294 (85.0%) reported doing an HIV test, and 178 (80.9%) reported never discussing HIV with their partners before. In addition, 220 (63.6%) were in a relationship, 175 (67.1%) had at least one sexual partner, and 100 (38.3%) used a condom in the last 6 months when having sexual intercourse. Furthermore, 58 (26.4%) knew their partner's HIV status and 277 (80.1%) did not think they were likely to get HIV. Among the sexual risk behaviors, condom use in the last 6 months when having sexual intercourse was significantly associated with willingness to use PrEP (*p* = 0.03).

**Table 2:** Cross‐tabulation: Sexual risk behavior indicators and willingness to use pre‐exposure prophylaxis (PrEP).

Risk indicator	Total population *n* (%)	Willingness to use PrEP		
		No, *n* (%)	Yes, *n* (%)	*p*‐Value
Are you in a relationship? *n* = 346				
No	126 (36.4)	103 (81.8)	23 (18.2)	0.65
Yes	220 (63.6)	184 (83.6)	36 (16.4)	
Have you ever had sex? *n* = 346				
No	85 (24.6)	76 (89.4)	9 (10.6)	0.07
Yes	261 (75.4)	211 (80.8)	50 (19.2)	
How many sexual partners do you have? *n* = 261				
1	175 (67.1)	143 (81.7)	32 (18.3)	0.61
>1	86 (32.9)	68 (79.1)	18 (20.9)	
In the last 6 months, did you use a condom when you had sex? *n* = 261				
No	161 (61.7)	126 (78.3)	35 (21.7)	0.18
Yes	100 (38.3)	85 (85.0)	15 (15.0)	
Have you been diagnosed with any STI in the past 3 months? *n* = 346				
No	341 (98.6)	283 (83.0)	58 (17.0)	>0.99
Yes	5 (1.5)	4 (80)	1 (20)	
Have you had a test for HIV in the last 3 months? *n* = 346				
No	52 (15.0)	46 (88.5)	6 (11.5)	0.25
Yes	294 (85.0)	241 (82.0)	53 (18.0)	
Do you know your partner's HIV status? *n* = 220				
No	162 (73.6)	135 (83.3)	27 (16.7)	0.84
Yes	58 (26.4)	49 (84.5)	9 (18.2)	
Do you discuss HIV with your partner? *n* = 220				
No	178 (80.9)	150 (84.3)	28 (15.7)	0.60
Yes	42 (19.1)	34 (81.0)	8 (19.0)	
How likely do you think you will get HIV? *n* = 346				
No	277 (80.1)	235 (84.8)	42 (15.2)	0.06
Yes	69 (19.9)	52 (75.4)	17 (24.6)	
When you had to take antibiotics or other medications, was it easy for you to remember to take the daily dose? *n* = 346				
No	175 (50.6)	150 (85.7)	25 (14.3)	0.17
Yes	171 (49.4)	137 (80.1)	34 (19.)	

*Note*: Results are frequency (%). The differences in proportions was assessed using Pearson Chi‐square test or Fisher's exact test as appropriate.

Abbreviation: PrEP, pre‐exposure prophylaxis.

Results from the multivariable regression analysis are presented in Table 3. In the unadjusted model, awareness of PrEP and never using a condom in the last 6 months when having sex were associated with the willingness to use PrEP. Never used a condom in the last 6 months when having sex was associated with 47% reduced odds of willingness to use PrEP (OR = 0.53, 95% CI 0.30, 0.94). On the other hand, awareness of PrEP was associated with increased odds of willingness to use PrEP (OR = 3.48, 95% CI 1.34, 9.07). After adjusting for other predictors, awareness of PrEP was associated with 3.03‐fold increase in the odds of willingness to use PrEP (AOR = 3.03, 95% CI 1.10, 8.40).

**Table 3:** Adjusted associations between predictors and willingness to use pre‐exposure prophylaxis (PrEP).

Variable	Willingness to use PrEP	
	COR (95% CI)	AOR (95% CI)
**Socio‐demographics**		
Age (years)		
24–29	1.77 (0.94,3.33)	1.19 (0.59, 2.37)
≥30	1.21 (0.56, 2.59)	0.80 (0.32, 1.98)
Year of Study		
Second	0.78 (0.22, 2.82)	
Third	1.09 (0.28, 4.27)	
Fourth	1.19 (0.36, 3.96)	
Fifth	2.48 (0.78, 7.89)	
Sixth	2.03 (0.50, 8.15)	
**Sexual risk behavior indicators**		
Ever had sex	2.01 (0.94, 4.27)	1.59 (0.66, 3.86)
Never used condom in last 6 months when had sex	**0.53** (0.30, 0.94)*	0.71 (0.36, 1.38)
Never discuss HIV with a partner	0.56 (0.28, 1.09)	0.58 (0.28, 1.22)
Don't think will get HIV	0.55 (0.29, 1.04)	0.70 (0.35, 1.39)
Not easy to remember the daily dose of medication	1.49 (0.84, 2.62)	1.49 (0.83, 2.66)
**Awareness of PrEP**	3.48 (1.34, 9.07)*	3.03 (1.10, 8.40)*

*Note*: Boldface indicates statistical significance (*p < 0.05).

Abbreviations: AOR, adjusted odds ratio; COR, crude odds ratio; HIV, Human immunodeficiency virus; PrEP, pre‐exposure prophylaxis.

**Figure 2:**
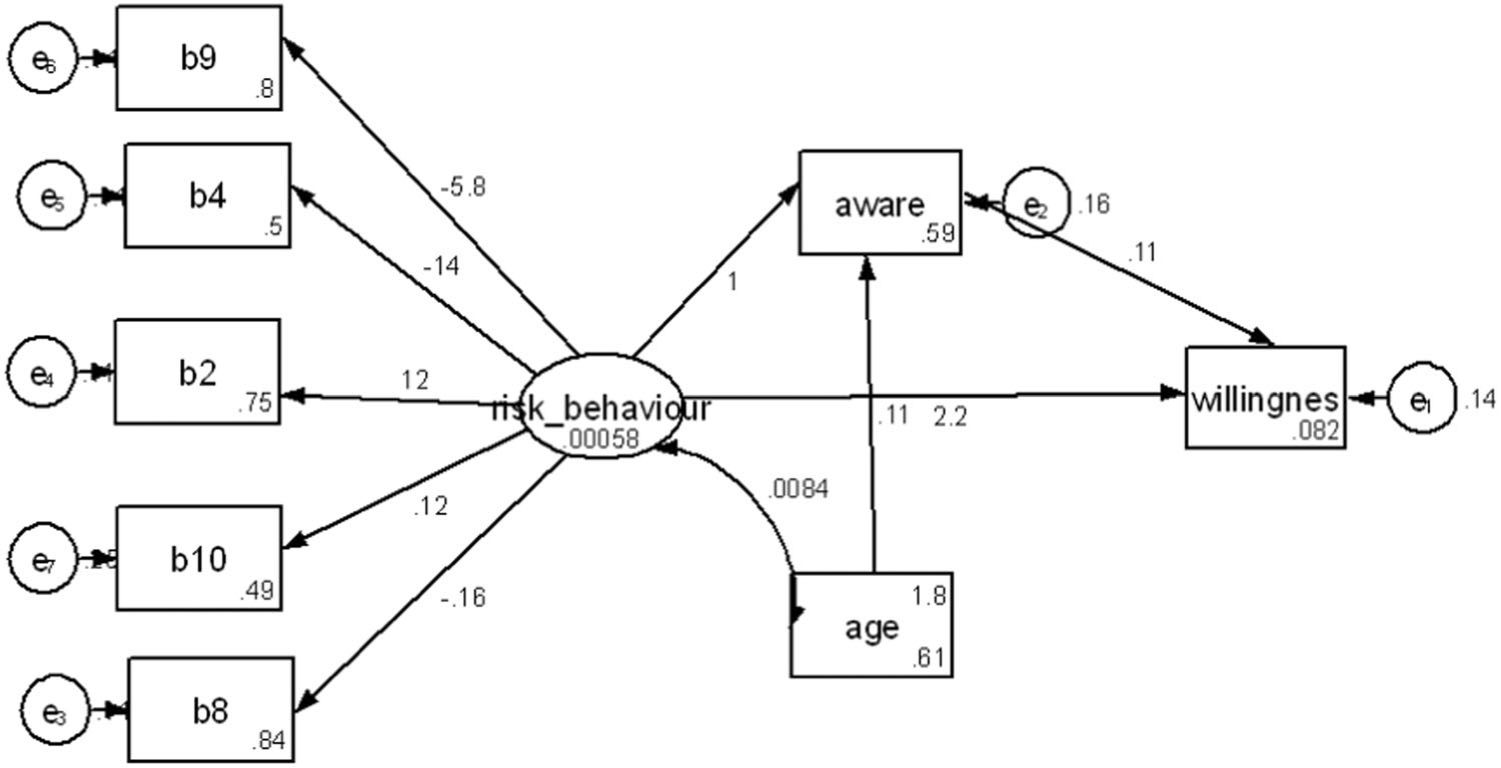
Structural equation model (SEM) of the relationship between sexual risk behavior and willingness to use PrEP (*n* = 346). Goodness of model fit Chi‐square = 30.3 (df = 18), *p* = 0.035; CFI = 0.931; RMSEA = 0.045 (90% CI = 0.012, 0.071). Standardized parameter estimates represent relationships. The boxes indicate measured variables, while the oval represents a latent variable. b1‐b10 are sexual risk behavior indicators. Aware‐awareness of PrEP, willingness‐willingness to use PrEP. Only pathways of age‐risk behavior and sexual risk behavior‐awareness‐willingness to use PrEP are significant at *p* < 0.05. RMSEA, root mean square error of approximation. CFI, comparative fit index (Figure 2).

In the SEM, sexual risk behavior increased willingness to use PrEP through some direct and indirect pathways (Figure 2). In the first pathway, sexual risk behavior had a mild effect on increased awareness of PrEP, leading to significant increases in willingness to use PrEP. In the second pathway, sexual risk behavior correlated with age, which altered awareness of PrEP and predicted increased willingness to use PrEP. In the third pathway, sexual risk behavior directly increases willingness to use PrEP, although random chance findings could not be ruled out in this pathway. The model fit was strong, with CFI = 0.931; RMSEA = 0.045

**Figure 3:**
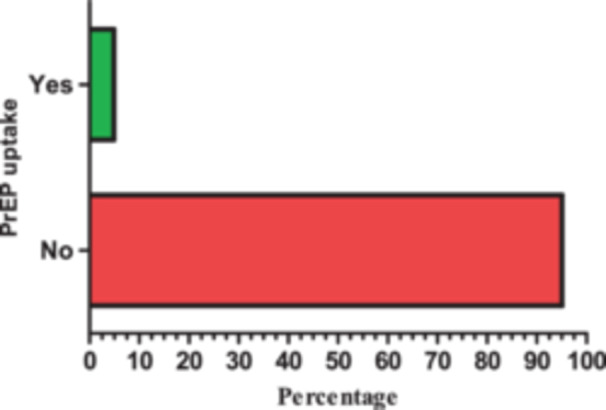
Pre‐exposure prophylaxis (PrEP) uptake among the participants (*n* = 346).

PrEP uptake among the participants: Regarding PrEP use among the participants, only 17 (4.9) were taking PrEP at the time of the study as shown in Figure 3.

## 6 DISCUSSION

This study aimed to explore the awareness and willingness to use PrEP among the students at UNZA. As one of the populations with the most significant incidence of HIV in the nation,^2^ university students were the study's primary focus. Even though most students were aware of PrEP, the study's findings revealed a low willingness to use PrEP among university students. Sexual risk behavior indirectly and positively affects willingness to use PrEP through the awareness of PrEP. Students who were aware of PrEP were more likely to use it as a preventive measure against HIV infection.

In this study, only 17.1% were willing to use PrEP if they were at risk of acquiring HIV infection. The proportion of students willing to use PrEP in our study is very low compared to other similar studies among adolescents and young adults.^23^, ^28^, ^29^, ^30^ We speculate that a lack of knowledge about the benefits of PrEP, as well as a poor understanding of HIV and AIDS prevention risks, could explain the observed results because one study found that these can negatively affect willingness to use PrEP.^28^ In contrast to our study, other studies have found that one's perception of oneself as being at high risk of HIV acquisition and having had an HIV test in the last 6 months influences one's willingness to use PrEP.^31^ This finding from the current study has significant implications for HIV prevention in Zambia, which is one of the most HIV‐affected countries in the Sub‐Saharan region.

It is critical to provide full HIV prevention packages to all students, including awareness and access to PrEP. In an adjusted logistic regression analysis, awareness of PrEP was significantly associated with willingness to use PrEP. This revelation was supported by the findings of a meta‐analysis study.^32^ The current study's low willingness to use PrEP necessitates raising awareness through online and print media as well as community health talks.

In this study, sexual risk behavior indicators and willingness to use PrEP revealed that a higher proportion of students who did not report condom use in the last 6 months were willing to use PrEP than those who reported condom use (21.7% vs. 15.0%). The plausible explanation for the current finding is that students who did not report condom use perceived themselves as being at high risk of HIV acquisition and, therefore, felt the willingness to use PrEP in the future. This finding is supported by studies done among students in Lesotho and Thai universities, which reported that students who disliked condoms were more likely to use PrEP than those who did report condom use.^23^, ^33^ In another study, students who reported being at high risk of HIV acquisition and strongly adhering to antibiotics were more willing to use PrEP.^23^ This is not supported by what was observed in our study, even though 80.1% of the students perceived themselves to be at risk of HIV acquisition.

The current study revealed that 78.3% of respondents exhibited awareness regarding PrEP. This level of awareness exceeds that documented among university students in previous studies.^23^, ^34^, ^35^ Conversely, our findings contrast with those of other research, which identified higher levels of PrEP awareness than ours.^34^, ^35^ A meta‐analysis study revealed that awareness of PrEP varies among nations according to factors such as economic standing, WHO regions, the type of PrEP (formulation and mode of administration), policies, publications, and the years the research was done.^32^ In this study, SEM showed that sexual risk behavior did not affect willingness to use PrEP independently, but awareness of PrEP was a modifying factor in this relationship. The possible explanation is that 75.4% of students who perceived themselves to be at higher risk of contracting HIV were not willing to use PrEP, suggesting that willingness to use PrEP is not always driven by higher HIV risk self‐perception.^36^ Contrary to our finding, one study showed that sexual risk behavior predicts willingness to use PrEP.^23^ This apparent inconsistency in our study may be explained by higher‐risk animosity and by the fact that certain personality traits, like opposition to condom use, are independently strong predictors of PrEP use, regardless of HIV risk. Therefore, in the future, campaigns aimed at increasing willingness to use PrEP should also pay attention to elements indirectly related to one's opinion of their HIV risk such as anxiety, perceived vulnerability, optimism bias, or fatalism which can all impact how individuals perceive their risk of HIV and their likelihood of taking preventive actions.

The current study revealed that only 4.9% of students were using PrEP at the time of the study. Our finding is supported by other studies that have reported low PrEP uptake among students.^23^, ^27^ The low PrEP uptake displayed in this study could be due to a lack of worry and a low willingness to use PrEP. Lack of worry about HIV was also highlighted by another study as a barrier to PrEP intake.^37^ In the present study, 80.1% of the respondents viewed themselves as not being at risk of contracting HIV infection. Therefore, this can also contribute to the low PrEP uptake observed in this study because some studies have shown a high uptake of PrEP among high‐risk populations such as sex workers and long‐distance truck drivers.^38^ Some studies have also revealed that PrEP usage may be influenced by age, as young people are at risk of using PrEP at lower rates than they require.^39^, ^40^ This might be supported by the present study, as the majority of the respondents were young.

When the participants were asked about their source of information on PrEP, the majority (45.4%) of participants in this study obtained the information from the health workers. This finding is supported by other studies.^41^, ^42^ Our results suggest that students view healthcare workers as the main source of information about PrEP. Therefore, there is a need for health workers in the country, through the Ministry of Health, to take an active role in developing innovative measures that promote the importance of PrEP awareness and uptake. In contrast, one study reported that those who had heard about PrEP learned about it most frequently from the media or online,^43^ but our study revealed that a small proportion (19.3%) of participants heard about PrEP from the media. Therefore, there is a need to increase the dissemination of PrEP information through Zambian social media platforms and through articles in newspapers and magazines to improve PrEP awareness, willingness, and subsequent uptake.

There were various limitations in this study. The study's restriction to university students may not accurately represent Zambia's broader adolescent population. Furthermore, the sample was only drawn from Lusaka, the capital, thereby limiting the scope of extrapolation to more rural communities. Another limitation was that all of the data was self‐reported; therefore, social desirability bias may have been encountered. However, questions were appropriately phrased, and the survey was completed in private. Furthermore, compared to similar studies, this study used SEM to assess direct and indirect pathways between risky sexual behavior and willingness to use PrEP, which is robust to latent variables.

## 7 CONCLUSION

We set out to explore the awareness of PrEP and the willingness to use it among the University of Zambia students. Although the awareness level of PrEP was high, most students were unwilling to use it, and only a small proportion were using it. This study also revealed that sexual risk behavior indirectly increased willingness to use PrEP through awareness of PrEP. There is, therefore, an immediate need to ramp up efforts to educate students about the importance of PrEP uptake as a preventive measure to reduce HIV transmission.

## AUTHOR CONTRIBUTIONS

**Martin Kampamba**: Conceptualization; Investigation; Methodology; Project administration; Resources; Formal analysis; Supervision; Writing—original draft; Writing—review and editing; Data curation. **Natalia N. Nelumbu**: Conceptualization; Methodology; Data curation; Investigation; Formal analysis; Supervision; Writing—review and editing; Writing—original draft; Resources; Project administration. **Christabel N. Hikaambo**: Resources; Software; Validation; Visualization; Writing—original draft; Methodology. **Steward Mudenda**: Methodology; Software; Validation; Visualization; Writing—original draft. **Jimmy M. Hangoma**: Investigation; Methodology; Writing—original draft. **Mwape Kunda**: Methodology; Investigation; Writing—review and editing. **Webrod Mufwambi**: Methodology; Investigation; Writing—review and editing. **Audrey Hamachila**: Methodology; Investigation; Writing—review and editing. **Moses Mukosha**: Conceptualization; Methodology; Project administration; Writing—review and editing; Writing—original draft; Investigation; Data curation; Formal analysis; Supervision; Resources.

## CONFLICT OF INTEREST STATEMENT

The authors declare no conflict of interests.

## ETHICS STATEMENT

The protocol for this study was approved by the University of Zambia School of Health Sciences Research Ethics Committee (Study protocol ID: 20203101050). We obtained informed, written consent from all participants before the study. The participants’ confidentiality and anonymity were strictly maintained. Informed consent from all the participants was taken.

## TRANSPARENCY STATEMENT

The lead author Martin Kampamba affirms that this manuscript is an honest, accurate, and transparent account of the study being reported; that no important aspects of the study have been omitted; and that any discrepancies from the study as planned (and, if relevant, registered) have been explained.

## Data Availability

The data that support the findings of this study are available from the corresponding author upon reasonable request. Upon reasonable request, the corresponding author, M.K., would provide the data that support the study's conclusions.
